# Supplementary figures and images for: A model of naturalistic decision making in preference tests
Source: PLoS Comput Biol. 2021 Sep 23;17(9):e1009012. doi: 10.1371/journal.pcbi.1009012 (PMC8491944; doi:10.1371/journal.pcbi.1009012)

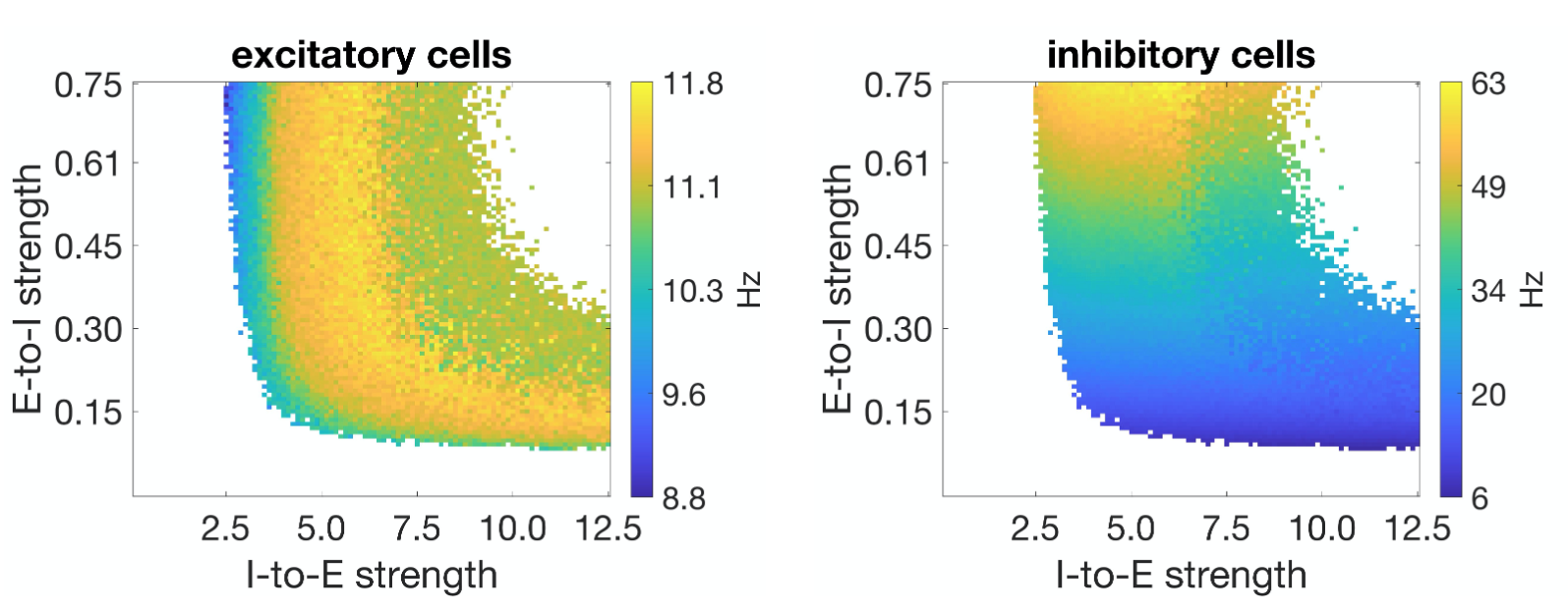

Supplement: S1 Fig — (Left) Firing rate of excitatory cells is in the vicinity of 10 Hz for all parameters leading to two quasi-stable network states. (Right) Firing rate of inhibitory cells varies strongly as a function of parameters, but note that the range of rates is similar for entice-to-stay networks (left edge) and repel-to-leave networks (right edge) so firing rate is not a clear indicator of type of network. (TIFF) [file pcbi.1009012.s001.tiff]

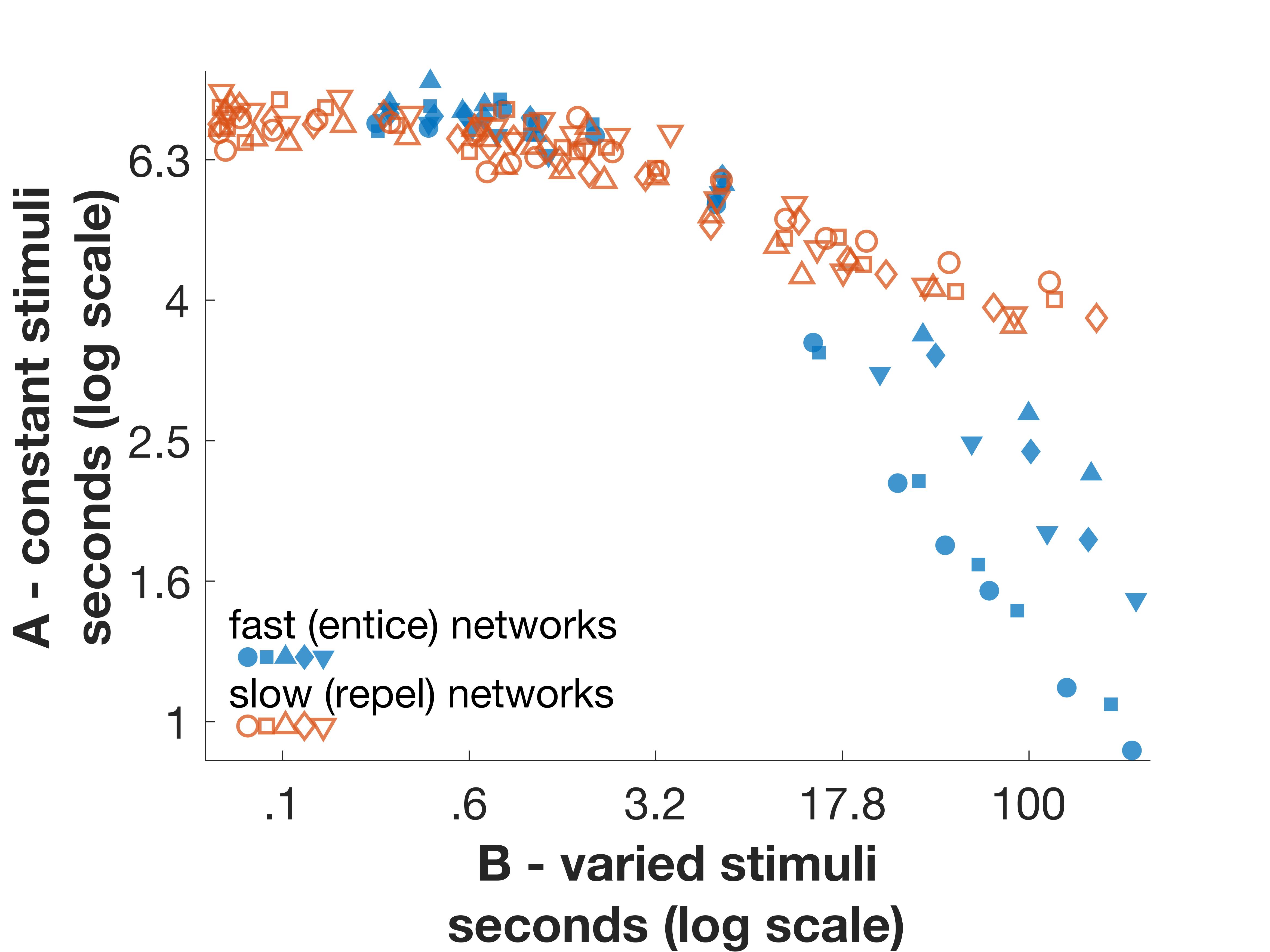

Supplement: S3 Fig — All network results are shown, with marker symbols identifying the network parameters as depicted in Fig 2 (main text Fig). Results for all of the intrinsically fast-switching (entice-to-stay) networks exhibited a stronger impact on the duration of bouts at the stimulus of fixed input, A, as the strength of input from the alternative stimulus, B, was adjusted to reflect more hedonic input. (TIFF) [file pcbi.1009012.s003.tiff]
