# Supplementary material for: A model of naturalistic decision making in preference tests
Source: PLoS Comput Biol. 2021 Sep 23;17(9):e1009012. doi: 10.1371/journal.pcbi.1009012 (PMC8491944; doi:10.1371/journal.pcbi.1009012)
Supplement: Attachment — Submitted filename: Response to reviewers.pdf [file pcbi.1009012.s005.pdf]

Response to Reviewers Comments

We thank both reviewers for their careful reading of the manuscript and positive comments. We have taken their critiques into account, in order to add some necessary clarification to the manuscript, updating figures and text accordingly. We respond to individual comments below.

Reviewer #1: The authors present a very interesting and clear set of simulations exploring switching and decision making networks. The methods are clear, results coherent and well introduced. What is lacking is a discussion as to how this applies to brain areas in general or specifically. The networks are simple feedback excitatory networks with global inhibition allowing to exhibit attractor states and to have these be suppressed. While each individual module could easily represent a cortical area or part of one, I don't know of any cortical areas that interact with each other by exciting the other networks inhibitory neurons (with the exception of olfactory cortex projections to olfactory bulb). I would encourage the authors to add some discussion on where and how these phenomena would be implemented either as a general or taste preference specific mechanism. As it is one is left with a rather simplistic analysis of mutual interactions between attractor networks. Which are very interesting in the context presented but need integration into the biological question.

Thank you for these supportive comments. We agree it is important to provide some indication of where the neural circuitry resides. We should point out that we were unclear in the original manuscript, in that we assume all neurons we model are in the same region of the brain. Cross-inhibition among groups of cells leading to mutually exclusive responses are common in models of decision making and premotor planning. So, although there has been some work suggesting prefrontal cortex projects to inhibitory neurons elsewhere as a form of inhibitory control (see e.g. work by Helen Barbas) we do not assume that is the case in our model. Rather we would assume prefrontal neurons with different outputs inhibit each other. In the text we have now added in the Discussion (l.343-347):

"While our model does not designate the location of the simulated circuit, we assume all neurons in our model are located in the same region and are likely to be found in anterior cingulate cortex, where neurons represent value in self-paced decisions [1], or a subregion of the ventromedial prefrontal cortex, such as the infralimbic cortex, whose activity is needed for an animal to engage in feeding behavior[2]."

[3]Reviewer #2: The present work addressed decision making processes in the interesting context of a preference task. The computational models are proposed to underlie two opposing strategies that animals might follow in taste preference tests: one in which the animal prefers to switch unless the current stimulus is highly appealing ('enticed to stay') and another in which the animal prefers to stay with its current choice, unless it is repelled from such choice by the attractiveness of other available options ('repel to leave'). The study of these models leads to behavioral and electrophysiological predictions to discern between both strategies.

I think that the study is quite original and tackles a highly interesting topic.

Thank you for the supportive comment.

I have however one concern that diminished my enthusiasm about the presented results (plus a few others that should also be addressed):

1) While the paper is overall well written, and the models themselves are standard spiking networks, the interpretation given to such models and their components is extremely hard to follow and at points inconsistent. This makes the paper really unclear and potentially misleading in its conclusions. Inconsistencies appear across several figures and results:

-In figure 1, we are presented with a first version of the model, in which one excitatory population encodes the option 'stay' (blue in the figure) and the other one encodes the option 'switch' (orange). Panel B shows that both populations can sustain their activity for several seconds --however, I have a hard time understanding 'switch' as a choice that is sustained for long periods, rather than a sharp signal triggering a motor action. What's the meaning of a populations which persistently encodes 'switch' over several seconds?

Thank you for pointing out the lack of clarity around Figure 1, which we have now replaced. Our original intent was to build up the model components step by step, but we realize now that showing a figure with neural activity that never appears in the conditions of normal practical operation of our model is both confusing and misleading. Instead, we have replaced Figure 1 with the model in normal operating conditions, in which the duration of the "switch" pool is more transient.

-Figure 3 then shows that the duration of the 'switch' option is extremely short, contradicting what the authors showed in Fig. 1. This is due, the authors explain later, to the fact that the simulation is reset after each switch. This makes sense as it prevents the 'switch' population from displaying sustained activity for long periods, although it makes the model discontinuous from one choice to the next.

We have now clarified the term "reset" which caused the later confusion as it sounds like a "hard reset" of a machine, which is not what we did. Rather we simply removed stimulus input to represent the animal moving away from the stimulus then, without modeling the movement and change of location explicitly, we subsequently reduced input to the "switch" pool to denote the end of the "switch" to a new stimulus. This ensured that when the animal initiates a sampling bout at a new stimulus the corresponding neurons in the "stay" pool become active to match that behavior.

Lines 197-200 now read:

"We do not simulate the behavior of the animal between bouts of tasting, but once the activity state indicates a switch of stimuli, we remove all stimulus input in the model to indicate movement away from the food source. We subsequently reduce input to the excitatory "switch"-producing cells to indicate completion of the movement and ensure commencement of the next sampling bout with the "stay" state."

-The work then focuses on the effects that the duration of a given choice ('stimulus A') has on the duration on the next chosen option (say, stimulus B). This is precisely one of the only things that the model, as explained up to Fig. 3, should be unable to do given the hard reset after a decision to switch.

We apologize for the prior lack of clarity and hope that our rewriting ensures that the loss of "switch" dominance is explained as something other than a "hard reset" following our rewriting of lines 197-200.

The authors reinterpret the model again to accommodate this continuous simulation of 1500 seconds, by effectively swapping the populations 'stay' and 'switch' after each switch.

Actually, we never swap the populations – it is unclear to us which part of our manuscript gave rise to such an interpretation. In for example, the "entice-to-stay" network, all stimulus input excites the same "stay" excitatory cells. These are always "stay" cells in the model and there is no swapping of populations. In fact, this is a key distinction of our model for preference tests – where stimulus input excites the same cells in a temporally separated manner – from models of perceptual decisions where distinct simultaneous stimuli activate distinct cell groups. We have now, in the introduction, added a paragraph to emphasize this key point of preference tests comparing the same "value" or hedonic or "palatability" signal across time (lines 76-85):

"Our simulations differ from those aimed to model the circuitry underlying perceptual decision making in a second way. In preference tests the stimuli are separated in time, rather than via distinct neural pathways. For example, two tastes being compared could simply be different concentrations of sodium chloride concentration, which yields an inverted-U palatability response[4]. The two stimuli excite identical neurons during successive sampling bouts, with neurons indicating high palatability during one bout being the same as those indicating high palatability during the next bout. In this manner, a preference task is more akin to decision making tasks requiring a sequential comparison of a single parametric quantity[3, 5, 6], although in this paper the quantity being compared is palatability and the decision is an ongoing one of how long to stay at the stimulus."

This is an extremely complicated way of simulating something much simpler: a competition between stimulus A and B which cross-inhibit each other (something that, on the other hand, is not as novel as the proposed topic in which behavioral conditions 'stay' and 'switch' are the opposing options rather than two input choices).

We hope we have clarified that given the stimuli are not concurrent and they activate the same pathway, both in practice and in our model, they would not cross-inhibit each other in this manner and we always have one population representing "stay" and the other representing "switch". Neurons whose activity is monotonic in the palatability of the stimulus (or other hedonic quality in the more general case) would respond and provide input to one group of cells, independently of the precise palatability-independent sensory details of the stimulus.

If the authors would like to model the competition between staying and switching for a taste

preference task, a much cleaner way would be to build a model with two well-defined excitatory populations (encoding taste 'A' and taste 'B') and a third population with sharp, transient responses for the 'switch' command. In the present framework, on the other hand, it is very unclear what each of the populations encodes along all the simulation, and arbitrarily swapping labels may invalidate results about inter-trial memory effects sought by the authors.

The review suggests a good alternative model, which could be appropriate in some tasks. We choose to stick with our model here, in part because it is simpler (in having only one "stay" and one "switch" pool) and has specific predictions already. Also, it is unclear how a simple model would, for example, target one concentration of salt solution to one memory pool and a subsequent one to the other memory pool – given the inputs are transduced by the same stimulus pathway. In order to gate inputs to different neural populations according to stimulus location, I think an extra assumption, or complicated pre-processing circuit would be needed, in contrast to our model in which successive inputs excite the same cells and indeed the motor response to "just keep licking" is the same indication of preference for each input.

Other comments:

2) While the authors did a good job taking into account the effects of variability in E-to-I and I-to-E synapses, it would be interesting to see whether the model is robust to physiologically plausible connections not present in the model, such as E-to-E (both recurrent and reciprocal) and I-to-I.

Our standard model does indeed have recurrent E-to-E connections (the curved arrows in Figure 1A). We have now added simulations which include the extra connections suggested (E-to-E between groups, as well as I-to-I within and between groups, all at 50% of the strength of E-to-E within-group connections) and produced a phase diagram indicating the robustness of the extended system in what is now Supplementary Figure 2. In this version, the results differ little from the original, so we choose to keep the original, simpler system with the dominant connections, as our main figure. In general, connections can either enhance or reduce competition and change in any one connection type can be compensated for by a change in another connection type. There appears to be a range of connection strengths such that the competition is appropriate to allow for the observed durations of states given appropriate stimuli.

3) Fig 1: panel D and C should be exchanged, as D is introduced and explained earlier in the text than C.

Thank you for the suggestion. We have fixed this.

4) Fig 2: aren't panels A and B the same? If so, authors could drop panel A, as the symbols in B are not really occluding much of the figure.

The reviewer is correct, so we have reduced to the single panel.

5) Page 10: This sentence needs a citation: "durations between eight and nine seconds. These are typical bout durations for a rat licking water from a spout".

Thank you for the suggestion. We have now added two citations which indicate ranges from a few seconds to several tens of seconds (depending on taste stimulus and the state of the animal) in the literature. (We initially referred to unpublished preliminary data, so have rewritten the sentence to reflect the published literature). Lines 179-180 now read:

"We therefore defined a neutral stimulus for each network, as one which produces mean "stay state" durations between eight and nine seconds, in the middle of the observed ranges of typical bout durations for a rat licking taste stimuli from a spout[7, 8]."

Interestingly a prior paper notes an exponential distribution of bout durations, which is suggestive of the noise-induced state transitions utilized by our model.

6) Page 17, lines 263-5: "intrinsically slow switching (entice-to-stay) networks (left) and intrinsically fast-switching (repel-to-leave) networks (right)". Should the fast switching network be the entice-to-stay, and the slow one the repel-to-leave?

Thank you for the careful review and noticing such an embarrassing lapse by us. We have now fixed this issue (now lines 269-270).

Have the authors made all data and (if applicable) computational code underlying the findings in their manuscript fully available?

Reviewer #1: Yes

*Reviewer #2: **No:** The code has not been available and there is no indication in the manuscript that it will be made available upon publication.*

The code is available on github. We now state so at the end of the Methods (lines) and include the link:[7] https://github.com/johnksander/naturalistic-decision-making

1. Wang S, Shi Y, Li BM. Neural representation of cost-benefit selections in rat anterior cingulate cortex in self-paced decision making. Neurobiol Learn Mem. 2017;139:1-10. Epub 2016/12/07. doi: 10.1016/j.nlm.2016.12.003. PubMed PMID: 27919831.
2. Riveros ME, Forray MI, Torrealba F, Valdes JL. Effort Displayed During Appetitive Phase of Feeding Behavior Requires Infralimbic Cortex Activity and Histamine H1 Receptor Signaling. Front Neurosci. 2019;13:577. Epub 2019/07/19. doi: 10.3389/fnins.2019.00577. PubMed PMID: 31316329; PubMed Central PMCID: PMCPMC6611215.

3. Brody CD, Hernandez A, Zainos A, Lemus L, Romo R. Analysing neuronal correlates of the comparison of two sequentially presented sensory stimuli. Philosophical transactions of the Royal Society of London Series B, Biological sciences. 2002;357(1428):1843-50. Epub 2003/03/11. doi: 10.1098/rstb.2002.1167. PubMed PMID: 12626017; PubMed Central PMCID: PMC1693076.
4. Sadacca BF, Rothwax JT, Katz DB. Sodium concentration coding gives way to evaluative coding in cortex and amygdala. Journal of Neuroscience. 2012;32:9999-10011. PubMed Central PMCID: PMCPMC3432403.
5. Machens CK, Romo R, Brody CD. Flexible control of mutual inhibition: a neural model of two-interval discrimination. Science. 2005;307(5712):1121-4. Epub 2005/02/19. doi: 10.1126/science.1104171. PubMed PMID: 15718474.
6. Miller P, Wang XJ. Inhibitory control by an integral feedback signal in prefrontal cortex: a model of discrimination between sequential stimuli. Proc Natl Acad Sci U S A. 2006;103(1):201-6. doi: 10.1073/pnas.0508072103. PubMed PMID: 16371469; PubMed Central PMCID: PMCPMC1324991.
7. Davis JD. Deterministic and probabilistic control of the behavior of rats ingesting liquid diets. Am J Physiol. 1996;270(4 Pt 2):R793-800. Epub 1996/04/01. doi: 10.1152/ajpregu.1996.270.4.R793. PubMed PMID: 8967409.
8. Monk KJ, Rubin BD, Keene JC, Katz DB. Licking microstructure reveals rapid attenuation of neophobia. Chem Senses. 2014;39(3):203-13. Epub 2013/12/24. doi: 10.1093/chemse/bjt069. PubMed PMID: 24363269; PubMed Central PMCID: PMCPMC3921893.
